# Glycocalyx Preservation and NO Production in Fatty Livers—The Protective Role of High Molecular Polyethylene Glycol in Cold Ischemia Injury

**DOI:** 10.3390/ijms19082375

**Published:** 2018-08-12

**Authors:** Alexandre Lopez, Arnau Panisello-Rosello, Carlos Castro-Benitez, René Adam

**Affiliations:** 1 INSERM U935, Université Paris-sud, Villejuif, 94800 Paris, France; arnau.panisello@iibb.csic.es (A.P.-R.); ccastrob@gmail.com (C.C.-B.); rene.adam@aphp.fr (R.A.); 2 Centre Hépato-Biliaire, Hôpital Universitaire Paul Brousse, Villejuif, 94800 Paris, France

**Keywords:** glycocalyx, liver, steatosis, cold storage, ischemia, polyethylene glycol

## Abstract

Improving the protection of marginal liver grafts during static cold storage is a major hurdle to increase the donor pool of organs. The endothelium glycocalyx quality of preservation influences future inflammatory and oxidative responses. One cellular pathway responsible for the formation of nitric oxide by endothelial cells is dependent on the stimulation of proteoglycans present in the glycocalyx. We investigated the impact of the glycocalyx preservation in static cold storage of fatty liver preserved in different preservation solutions on the endothelium-mediated production of NO. Zucker fatty rat livers were preserved 24 h in static cold storage in either Institut Georges Lopez-1 (IGL-1) (*n* = 10), IGL-0 (i.e., without PEG35) (*n* = 5) or Histidine-Tryptophan-Ketoglutarate (HTK) (*n* = 10) preservation solutions before being processed for analysis. For Sham group (*n* = 5), the fatty livers were immediately analyzed after procurement. The level of transaminases and nitrites/nitrates were measured in the washing perfusate. Glycocalyx proteins expressions, Syndecan-1, glypican-1 and heparan sulfate (HS), were determined in the tissue (ELISA). Steatotic livers preserved 24 h in IGL-1 preservation solution have a significant lower level of transaminases (aspartate aminotransferase (AST), alanine aminotransferase (ALT)) and less histological damages than steatotic livers preserved 24 h with HTK (*p* = 0.0152). The syndecan-1 is significantly better preserved in IGL-1 group compared to HTK (*p* < 0.0001) and we observed the same tendency compared to IGL-0. No significant differences were observed with glypican-1. HS expression in HTK group was significantly higher compared to the three other groups. HS level in IGL-1 was even lower than IGL-0 (*p* = 0.0005) which was similar to Sham group. The better protection of the glycocalyx proteins in IGL-1 group was correlated with a higher production of NO than HTK (*p* = 0.0055) or IGL-0 (*p* = 0.0433). IGL-1 protective mechanisms through the formation of NO could be due to its better protective effects on the glycocalyx during SCS compared to other preservation solutions. This beneficial effect could involve the preservation state of syndecan-1 and the internalization of HS.

## 1. Introduction

The shortage of organs and the decrease in the quality of these organs are becoming an increasingly important public health problem in developed countries for the liver transplantation. One solution to increase the donor pool is to use marginal grafts such as steatotic livers. The use of these marginal grafts for liver transplantation could represent a way to increase the donor pool and thus to reduce the number of patients on the waiting list. The criteria to transplant or not marginal grafts are not very clear and each transplantation center takes its responsibility to choose the right graft for the right donor. During transplantation, cold ischemia time (CIT) needs to be reduced as much as possible in order to not cause irreversible damages to the liver [1]. This is especially true for fatty livers which are much more sensitive to static cold storage (SCS) compared to non-steatotic livers. Indeed, the availability of oxygen in steatotic livers is reduced due to an abnormal morphological change of the hepatocytes. The round-shape and swollen phenotype of these fat-loaded hepatocytes will narrow the sinusoidal lumen reducing the sinusoidal perfusion [2]. Thus, the understanding of the underlying mechanisms behind the steatosis is of utmost importance to improve the quality of the preservation and to reduce the postoperative complications for these organs.

The vascular endothelium can adapt to hemodynamic changes occurring in the blood vessels by regulating the vascular tone and structure. The nitric oxide (NO), as a mediator of vascular remodelling, is already described to play a main role in these mechanisms [3,4]. The NO is a molecule mainly synthesized by the constitutive endothelial nitric oxide synthase (eNOS) through the L-arginine, but it can also be produced by the inducible nitric oxide synthase (iNOS) present in the cytoplasm of some inflammatory cells which is upregulated during acute hepatic ischemia [5,6]. The genetic over expression or the prolonged delivery of NO reduce ischemia–reperfusion injury (IRI) in mice [7,8]. However, its functions in human liver transplantation are not clear. Some evidence has shown that a decrease in NO expression could exacerbate IRI in human orthotopic liver transplantation (OLT) [9,10] and could help to improve the hepatic microcirculation in OLT [11].

The endothelial glycocalyx is composed of proteoglycans (mostly syndecans and glypicans) and glycoproteins (heparan sulfate) bound to the luminar membrane of the endothelial cells. Glycocalyx thickness is linked with the shear stress in its local environment and can adapt to higher shear stress by remodelling the cells actin cytoskeleton [12]. IRI has already been identified to induce a very important degradation of the glycocalyx, demonstrating by the increase of syndecan-1 and heparan sulfate (HS) concentrations in the plasma of patients with end-stage liver diseases after surgery [13].

One of the main proteoglycans of the glycocalyx, the syndecan-1, has a cytoplasmic domain anchored to the cell surface through a glycosylphosphatidylinositol anchor and interacts with the actin cytoskeleton. It was postulated that syndecan-1 was the one responsible for the transduction of the physical signal from the blood flow to the cell leading to the synthesis of NO. However, it was first demonstrated by short-hairpin and more recently by selective atomic force microscopy pulling that the mechano-production of NO in response to shear stress is due to the glypican-1 and not the syndecan-1 [14,15].

Preservation solutions are commonly used to wash the organ during procurement and preserve it during transport. IGL-1 is known to be an efficient solution for normal but also for steatotic livers [16]. Several papers demonstrated that this benefit could come from the addition of polyethylene glycol 35 kDa (PEG35), an inert and non-toxic oncotic agent, which is the major difference with other commercial preservation solutions [17,18]. In our study, we compared the efficacy of IGL-1 with another preservation solution, HTK. The United Network for Organ Sharing (UNOS) and the European Liver Transplant Registry (ELTR) pointed out that the use of HTK was associated with an increased risk of graft loss in Donation after Circulatory Death (DCD) allografts and those with a cold ischemia time >8 h (UNOS) or a total ischemia time >12 h (ELTR) [19,20].

In our model, we tried to replicate the clinical conditions of prolonged CIT. To do this, we preserved the steatotic grafts for 24 h in SCS with the already available preservation solutions IGL-1, HTK or with a IGL-1-modified solution without PEG35, called IGL-0, to investigate the potential beneficial effects of the PEG35 in the grafts preservation. We measured the transaminases (AST and ALT) as well as the histological damage which are routinely assessed in clinic to assess the injury sustained by the livers during the SCS. Furthermore, we quantified the protein level of syndecan-1, glypican-1, HS to evaluate the preservation state of the glycocalyx and the correlation with the production of NO (the glycocalyx-mediated NO production pathway).

## 2. Results

### 2.1. Fatty Livers Injury and Histology after Cold Ischemia

**Figure 1:**
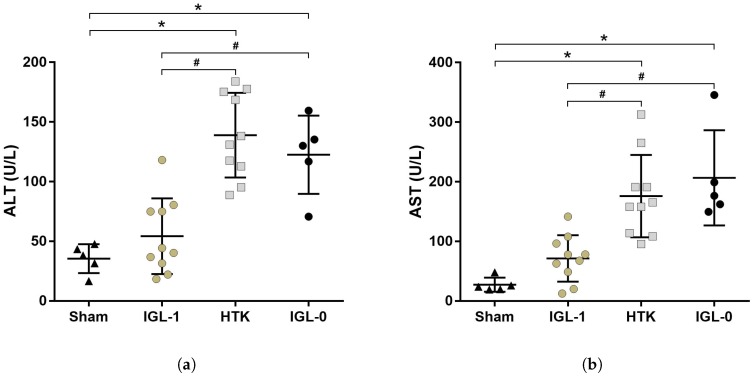
Fatty liver ischemia injury. Transaminases were collected after procurement (Sham group) or after 24 h of cold preservation in IGL-1, HTK or IGL-0 solutions (IGL-1, HTK and IGL-0 group, respectively). Transaminases ALT (**a**) and AST (**b**) release were higher in the HTK group compared to the three groups and IGL-0 transaminases were higher than Sham and IGL-1 groups. Results are presented as mean (SD), IGL-1/HTK *n* = 10/group, Sham/IGL-0 *n* = 5/group, * *p* < 0.05 vs. Sham; # *p* < 0.05 vs. IGL-1 (One-way ANOVA).

Level of transaminases (ALT, AST) were measured in the perfusate coming out from the inferior vena cava during the washing of fatty livers directly after procurement (Sham group) or after 24 h of cold storage in IGL-1, HTK or IGL-0 preservation solutions (IGL-1, HTK and IGL-0 group respectively) to assess the IRI sustained by the fatty livers due to cold ischemia (Figure 1).

Both transaminases levels in Sham groups are significantly lower compared to the transaminases of fatty livers preserved 24 h in HTK (AST: *p* = 0.007; ALT: *p* < 0.0001) and IGL-0 (AST: *p* = 0.0019; ALT: *p* = 0.0002) preservation solutions (Figure 1a,b). The transaminases level in IGL-1 group is lower than HTK (AST: *p* = 0.0149; ALT: *p* < 0.0001) and IGL-0 (AST: *p* = 0.0029; ALT: *p* = 0.0009) groups.

**Figure 2:**
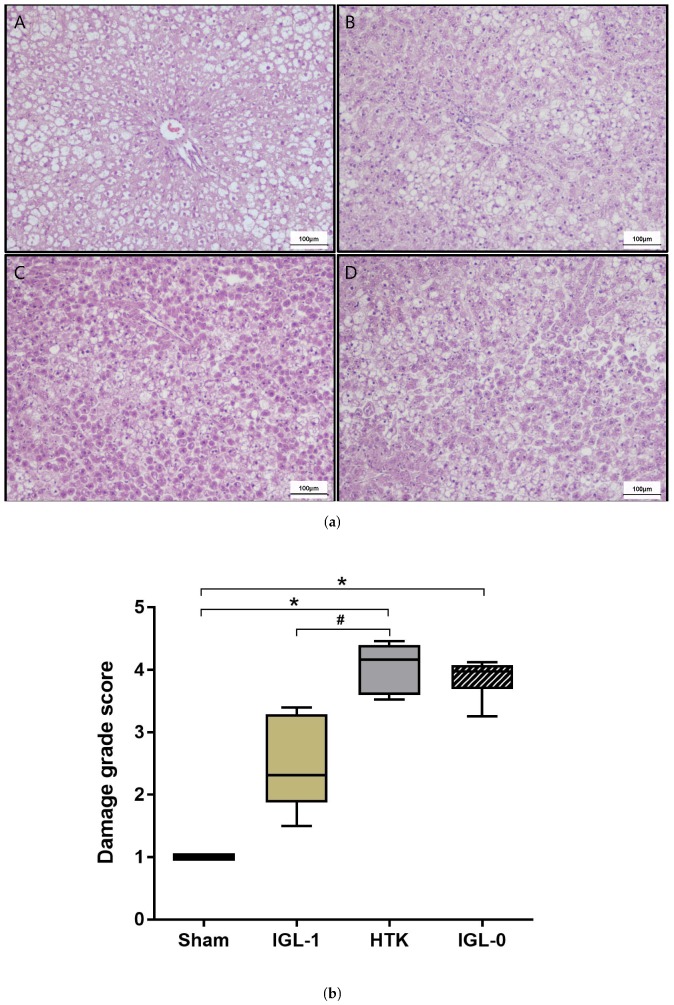
Fatty livers histology after cold ischemia. (**a**) Tissue samples were stained with HES (×10). Using Sham group (**A**) as a reference, IGL-1 group (**B**) showed less histological injury (i.e., cell dissociation, loss of hepatic architecture, swollen hepatocytes) than HTK (**C**) and IGL-0 (**D**) groups. (**b**) Damage grade score was made according to the preservation state of the samples evaluated by a pathologist (**E**).* *p* < 0.05 vs. Sham; # *p* < 0.05 vs. IGL-1 (Kruskal–Wallis test).

Tissue samples were stained with hematoxylin/eosin/safran (HES) to evaluate the cellular architecture preservation during prolonged cold ischemia (Figure 2). The histological assessment of lesions shows a severe degree of injury in HTK group compared to Sham group (*p* < 0.0001) with marked cell dissociation and loss of hepatic architecture, as well as numerous swollen hepatocytes (cell edema) (Figure 2(a-A,a-C)). In the IGL-1 and IGL-0 groups, although there was an expansion of sinusoids in extensive areas, the tissue preserved its structure with a mild to moderate cell swelling (Figure 2(a-B,a-D)). Accordingly, the damage grade score of IGL-1 group had a tendency to be lower compared to IGL-0 (*p* = 0.0584) and was significantly lower compared to HTK (*p* = 0.0152) groups but still higher than the Sham group (Figure 2b).

### 2.2. Syndecan-1, HS and Glypican-1 Preservation during Static Cold Storage

**Figure 3:**
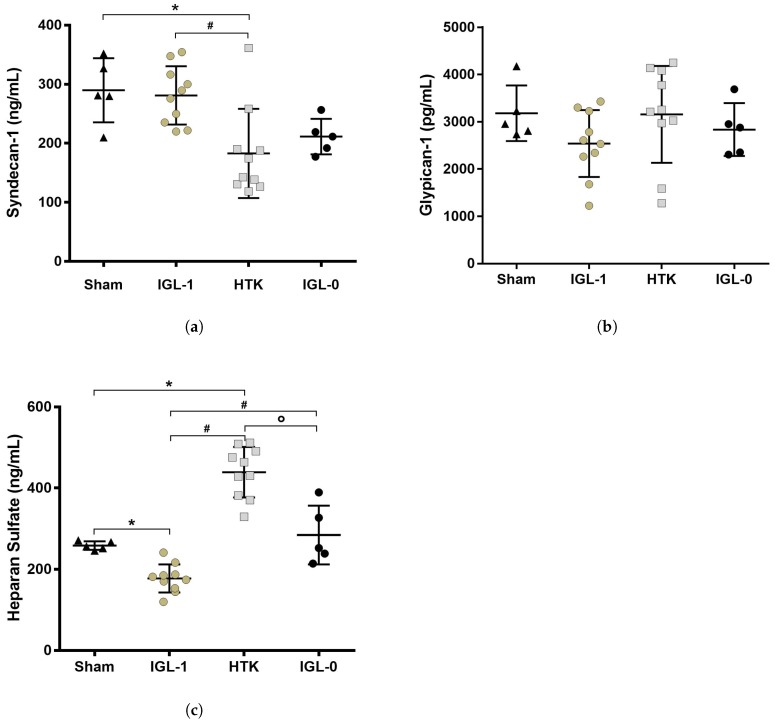
Glycocalyx protection in fatty livers after 24 h of cold storage. Protein expression of the three main components of the glycocalyx were measured by ELISA from tissue samples. (**a**) Syndecan-1 level was lower in HTK group compared to Sham and IGL-1 groups; (**b**) glypican-1 expression was the same in all the groups; (**c**) Heparan sulfate expression was lower in IGL-1 group compared to the three other groups and the opposite for HTK which expressed a higher level of HS. Results are presented as mean (SD), IGL-1/HTK *n* = 10/group, Sham/IGL-0 *n* = 5/group, * *p* < 0.05 vs. Sham; # *p* < 0.05 vs. IGL-1; ° *p* < 0.05 vs. HTK (One-way ANOVA).

IGL-1 efficiency to protect steatotic grafts has already been proven compared to other preservation solutions [20]. The PEG35, as the major components of IGL-1, could be the key for the higher protection of the grafts. The high molecular PEG35 has been reported to act as an endothelial cells barrier to prevent inflammatory events in acute lung injury [21]. Since the endothelial surface layer is composed of the glycocalyx, we quantified the syndecan-1, HS and the glypican-1, three main constituents of the glycocalyx, in order to evaluate and compare the integrity of the glycocalyx in fatty livers after being preserved 24 h in IGL-1, IGL-0 or HTK solutions (Figure 3).

After 24 h of SCS, Sham and IGL-1 groups did not have a different level of syndecan-1 expressed in their tissues. However, the HTK group contained significantly less syndecan-1 compared to Sham (*p* < 0.0034) and IGL-1 (*p* < 0.0018) groups (Figure 3a). Glypican-1 expression in tissues was the same in all four groups (Figure 3b). Interestingly, steatotic livers preserved in HTK solution for 24 h expressed more HS than all three groups (vs. Sham *p* < 0.0001; vs. IGL-1 *p* < 0.0001; vs. IGL-0 *p* = 0.002) (Figure 3c). It was the opposite for the IGL-1 group, which showed a decrease in HS expression compared to the other three groups (Sham *p* = 0.0274; vs. HTK *p* < 0.0001; vs. IGL-0 *p* = 0.0005). The HS level in the IGL-0 group was similar to Sham.

### 2.3. IGL-1 Protection through NO

It has been recently demonstrated that the production of NO by the endothelium is triggered by the glypican-1, even though this proteoglycan does not have a transmembrane domain like syndecan-1 [15]. Moreover, IGL-1 capacity to enhance NO production in steatotic livers was already demonstrated [22]. Thus, we measured the total quantity of NO_2_^−^ and NO_3_^−^ of the steatotic livers after 24 h of cold ischemia to investigate if the better protection of the glycocalyx is correlated with a higher level of NO in our samples.

**Figure 4:**
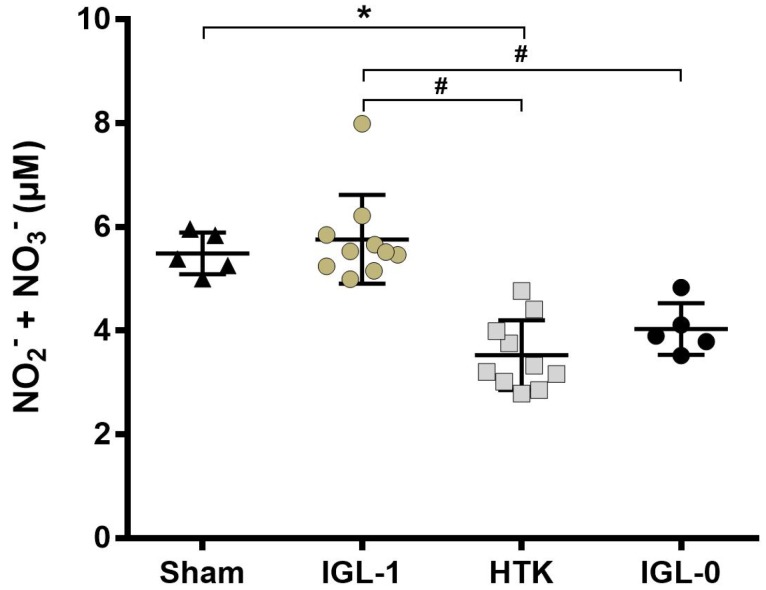
Nitrites/Nitrates expression after 24 h cold ischemia in steatotic livers. NO_2_^−^/NO_3_^−^ concentration was measured using a kit. HTK group production of NO_2_^−^/NO_3_^−^ was greatly reduced compared to Sham and IGL-1. The same decrease in the production of NO was observed when the PEG35 was removed (IGL-0) compared to IGL-1. NO_2_^−^/NO_3_^−^ in fatty liver preserved 24 h in IGL-0 was almost double compared to the Sham group. Results are presented as mean (SD), IGL-1/HTK *n* = 10/group, Sham/IGL-0 *n* = 5/group, * *p* < 0.05 vs. Sham; # *p* < 0.05 vs. IGL-1 (One-way ANOVA).

While steatotic livers expressed a lower level of NO_2_^−^/NO_3_^−^ after 24 h of static preservation in HTK solution compared to Sham (*p* < 0.0162) and IGL-1 (*p* < 0.0055), NO_2_^−^/NO_3_^−^ in the IGL-0 group was also decreased compared to the IGL-1 group (*p* < 0.0433) (Figure 4). This result further supports the positive regulation of NO by the IGL-1 solution.

## 3. Discussion

A better preserved graft for the liver transplantation is essential to reduce the IRI after reperfusion. This is even more true for sensitive organs such as steatotic livers. A simple way to optimize this preservation is the choice of preservation solution. Our study aimed to evaluate the preservation state of the glycocalyx during IRI in fatty livers subjected to a prolonged period of cold ischemia (24 h) in three different preservation solutions (IGL-1, HTK and IGL-0) and to investigate the role of the high molecular polyethylene glycol 35 kDa (PEG35) on this preservation and by extension its efficiency on the glycocalyx-mediated NO production.

We observed a better protective effect of IGL-1 compared to HTK with a lower level of enzymes release (transaminases) but also a better conservation of the hepatic tissues (Figure 1 and Figure 2a). The removal of the PEG35 from the IGL-1 solution induces a loss of these protective properties. These results tend to confirm the fact that HTK is not an optimal solution for preservation of liver steatotic grafts with a prolonged cold ischemia time and that the efficacy of IGL-1 partly comes from the oncotic agent PEG35.

The glycocalyx plays the role of a barrier to prevent leukocytes from adhering and therefore regulating the inflammatory events that can occur during IRI [23]. The alteration of glycocalyx components has been observed during heart surgery but also in patients with end-stage liver diseases during OLT [13,24,25]. The glycocalyx shedding is associated with a lot of negative effects on the organ (oedema, loss of endothelium-dependent vascular responsiveness, and enhanced adhesion of leukocytes and platelets). Thus, the integrity of the glycocalyx after SCS could be of utmost importance for the vascularization of the grafts. This could be even more true for the sensitive steatotic livers as demonstrated on steatotic grafts subjected to subnormothermic machine perfusion which had a perturbed microcirculatory state in case of glycocalyx dysfunction [26]. Moreover, the glycocalyx is known to be involved in the production of NO by the endothelial cells. The vasodilatation and antioxidant properties of NO play a key role in the protection of steatotic livers which are more exposed to microcirculation problems [27,28,29]. Supplementation of NO during the transplantation has been proposed to be used routinely in clinic to reduce IRI [30]. In our study, we confirm the upregulating effect of IGL-1 solution on NO synthesis in steatotic livers after prolonged cold ischemia that could be due to the presence of PEG35 as an inducible agent. The signalling pathway for the production of NO is linked with the internalization of the glypican-1 protein and its HS chains. The HS will be cleaved and used by the cells to further increase the production of NO [31]. This internalization of the glypican-1 is done through the caveolin-dependent endocytosis which are mostly localized in lipid rafts and are a preferential site for the enzyme responsible for the constitutive formation of the endothelial NO, eNOS [32,33,34]. eNOS activation depends on different experimental conditions of shear stress and it has been shown that its effect was mediated by glypican-1 [15,35].

**Figure 5:**
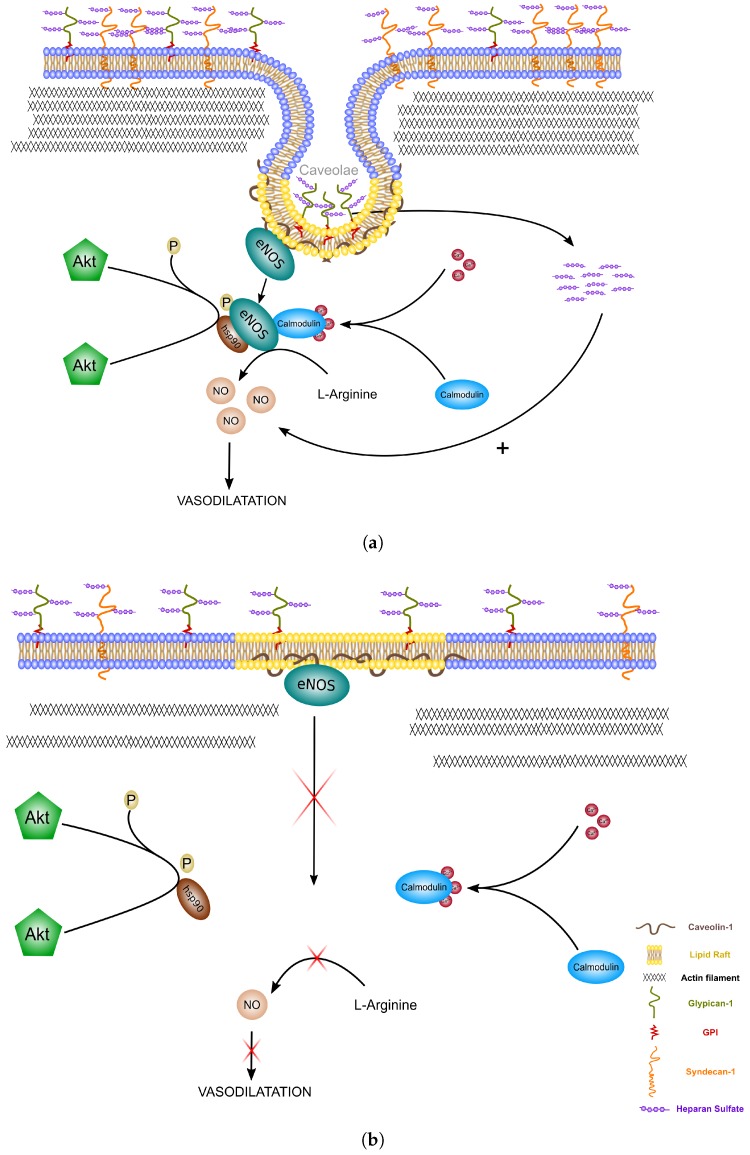
Endothelial glycocalyx-mediated NO production. (**a**) Internalization of the glypican-1 in caveolae is possible thanks to the stabilization of the actin filament by the transmembrane syndecan-1. The heparan sulfate is then cleaved to further increase the production of NO in the cells. This glycocalyx-mediated NO induces a vasodilatation of the endothelium and improves the steatotic graft condition; (**b**) in a graft with higher cold ischemia injury, the lower quantity of syndecan-1 is not sufficient to stabilize the cytoskeleton, which inhibits the formation of caveolae, leading to the absence of NO production.

In our experimental model, while we didn’t observe any significant differences for the expression of glypican-1, the steatotic livers preserved with HTK suffered a significant loss of the syndecan-1. Syndecan-1 has a transmembrane domain directly interacting with the cytoskeleton (i.e., actin filaments) and contributes to the remodelling of the cells [14]. The interaction of syndecan-1 with the actin filament helps to stabilize the caveolae formation as shown in Figure 5a. Thus, a reduction in syndecan-1 at the membrane of the cells could greatly reduce the number of caveolae formed (Figure 5b). eNOS will not be able to come into contact with the activating co-factors needed for its activation and, as a consequence, the production of NO will be impaired. Thus, the NO will not be able to perform its functions (i.e., the blood pressure regulation, vasodilatation, antithrombotic activity). These results were correlated with a lower expression of HS in the IGL-1 group compared to all the other groups. The degradation of these chains negatively affects the NO upregulation by the glycocalyx [36]. After 24 h of preservation in IGL-1 solution, it is highly possible that the HS chains are metabolised to increase the quantity of NO molecules produced by the endothelial cells, which could explain the lower expression of HS in this condition. However, the formation of caveolae could be impaired in steatotic livers preserved in HTK solution. Because of the decrease in syndecan-1, the HS can not be internalized and stay present on the cell membrane linked to the glypican-1, leading to a decrease of the activation of eNOS and the production of NO. The supplementation of HS could not be sufficient to induce NO synthesis and the preservation of the caveolin-endocytosis mechanisms could be of utmost importance. The addition of high molecular PEG has been shown to stabilize the lipid rafts under stress conditions and to remodel the actin cytoskeleton, which increase the cell–cell junctions and could further improve the glycocalyx-mediated NO production in livers preserved in IGL-1 solution [21,37]. The simple addition of PEG to preservation solutions could potentially protect against stress conditions such as IRI.

Our study provides further evidence on the benefit of IGL-1 solution and the NO and highlights the importance of protecting the glycocalyx, especially syndecan-1, in steatotic livers. In our knowledge, this is the first time that the concept of glycocalyx disruption during static cold preservation is suggested. To deepen our understanding of the physiological mechanisms implicated in the production of NO through glypican-1, future work should aim to study the internalization of glypican-1 and the involvement of the glypican-caveolin route in the glycocalyx-mediated NO production pathway in hepatic IRI. Moreover, in this study, the livers were not exposed to shear stress. To further explore the role of the glycocalyx in IRI, future studies should take into account the shear stress during the reperfusion of the organ and its effect on the glycocalyx integrity. Machine perfusion represents an interesting tool to investigate this.

## 4. Materials and Methods

### 4.1. Animals

Homozygous (obese [Ob]) Zucker rats aged 10–12 weeks were purchased from Iffa-Credo (L’Abresle, France). An “ex vivo” perfused rat liver model was used, as previously described. All procedures were performed under isoflurane inhalation anaesthesia. This study adhered to European Union regulations (EC-guideline 86/609/CEE) and approved by the Ethics Committees for Animal Experimentation (CEEA) of the University of Barcelona (number 483116; approved on 14 July 2016).

### 4.2. Liver Procurement and Experimental Groups

Liver procurement was performed as previously described [38]. The following experimental groups were defined as follows.

Zucker rats fatty livers were either preserved 24 h in static cold storage using IGL-1^®^ (n=10), HTK^®^ (n=10) or IGL-0 (n=5) preservation solutions or directly stored at −80 °C after procurement without any cold storage (Sham group, n=5). After cold storage, the liver were flushed with 20 mL of Ringer’s Lactate solution. The tissue samples were frozen in nitrogen and then kept at −80 °C for further analysis.

### 4.3. Biochemical Determination

#### 4.3.1. Histology

Liver samples were fixed in 10% paraformaldehyde and 3 µm sections were stained with hematoxylin/eosin/safran according to standard procedures. Damage grade score was assessed by a pathophysiologist taking into account several criteria such as lipid infiltration, cellular and sinusoidal dissociation, loss of cell architecture and cell edema.

#### 4.3.2. Transaminases Assay

AST and ALT were quantified using a commercial kit from RAL, Barcelona, Spain.

#### 4.3.3. Nitric Oxide Assay

NO production in liver was determined by tissue accumulation of nitrite and nitrate using a colorimetric assay kit (Cayman, Tallinn, Estonia).

#### 4.3.4. ELISA

Quantification of Syndecan-1 (Rat SDC1, E-EL-R0996, Elabscience, Houston, Texas, USA), Heparan Sulfate (Heparan Sulfate ELISA kit, OKEH02552, Aviva Systems Biology, San Diego, CA, USA) and glypican-1 (Rat GPC1, CSB-EL009703RA, Cusabio, Houston, TX 77036, USA) in tissue samples were obtained according to the manufacturer’s instructions. Optical density was quantified using a Modulus II Microplate Reader (Turner Biosystems, Sunnyvale, CA, USA).

### 4.4. Statistics

Data from the damage grade score were compared statistically by non-parametric ANOVA I (Kruskal–Wallis test) with Dunn’s post hoc analysis. All of the other data are expressed as means (SD), and were compared statistically by an ANOVA Type I test with post hoc Tukey Test for multiple comparison (Graph Pad Prism software, Inc., La Jolla, CA, USA). *p* < 0.05 was considered significant.

## 5. Conclusions

To improve the preservation conditions of grafts is of utmost importance in the near future and especially for those more vulnerable to IRI, as steatotic grafts. Measuring the main constituents of glycocalyx (at least syndecan-1 and heparan sulfate) to evaluate its preservation during the transplantation could potentially be an easy and reliable way to monitor the graft preservation state.

## Abbreviations

AST	Aspartate Aminotransferase
ALT	Alanine Aminotransferase
CIT	Cold Ischemia Time
ELTR	European Liver Transplant Registry
eNOS	endothelial Nitric Oxide Synthase
HTK	Histidine–Tryptophane–Ketoglutarate
HS	Heparan Sulfate
IGL-1	Institut Georges Lopez-1
iNOS	inducible NOS
IRI	Ischemia–Reperfusion Injury
NO	Nitric Oxide
OLT	Orthotopic Liver Transplantation
PEG35	PolyEthyene Glycol 35 kDa
SCS	Static Cold Storage
UNOS	United Network for Organ Sharing
